# Foreign DNA detection in genome-edited potatoes by high-throughput sequencing

**DOI:** 10.1038/s41598-023-38897-x

**Published:** 2023-08-09

**Authors:** Shuhei Yasumoto, Toshiya Muranaka

**Affiliations:** 1 Department of Biotechnology, Graduate School of Engineering, Osaka University, 2-1 Yamadaoka, Suita, Osaka 565-0871 Japan; 2 Industrial Biotechnology Initiative Division, Institute for Open and Transdisciplinary Research Initiatives, Osaka University, 2-1 Yamadaoka, Suita, Osaka 565-0871 Japan

**Keywords:** Biological techniques, Biotechnology, Computational biology and bioinformatics, Plant sciences, Plant biotechnology, Plant breeding

## Abstract

Genome editing is a powerful breeding technique that introduces mutations into specific gene sequences in genomes. For genome editing in higher plants, nucleotides for artificial nuclease (e.g. TALEN or CRISPR-Cas9) are transiently or stably introduced into the plant cells. After the introduction of mutations by artificial nucleases, it is necessary to select lines that do not contain the foreign nucleotides to overcome GMO regulation; however, there is still no widely legally authorized and approved method for detecting foreign genes in genome-edited crops. Recently, *k*-mer analysis based on next-generation sequencing (NGS) was proposed as a new method for detecting foreign DNA in genome-edited agricultural products. Compared to conventional methods, such as PCR and Southern hybridization, in principle, this method can detect short DNA fragments with high accuracy. However, this method has not yet been applied to genome-edited potatoes. In this study, we evaluated the feasibility of *k*-mer analysis in tetraploid potatoes by computer simulation, and also evaluated whether the *k*-mer method can detect foreign genes with high accuracy by analyzing samples of genome-edited potatoes. We show that when NGS data (at a depth of × 30 the genome size) are used, the *k*-mer method can correctly detect foreign genes in the potato genome even with the insertion of DNA fragments of 20 nt in length. Based on these findings, we expect that *k*-mer analysis will be one of the main methods for detecting foreign genes in genome-edited potatoes.

## Introduction

Genome editing has received interest from many researchers and breeders in plant sciences because it enables the addition of novel agronomic traits to crops. Genome editing using site-specific nucleases such as transcription activator-like effector nucleases (TALENs) and clustered regularly interspaced short palindromic repeat (CRISPR) is attracting attention as a novel plant breeding technology. Site-specific nuclease recognizes and introduces DNA double-strand breaks at specific sequences in the target genome, and the cleaved DNA is repaired by endogenous cellular mechanisms. However, repair errors can introduce mutations into the target DNA sequence. When new traits are created by genome editing, the genes required for the expression of site-specific nuclease enzymes are introduced into the target plants by *Agrobacterium*-mediated transformation or particle gun methods.

Organisms with exogenous nucleotides (or foreign genes) are treated as genetically modified organisms (GMOs) or living modified organisms (LMOs) in several countries, including Japan. The use of GMOs is regulated by strict laws, which create significant barriers to their commercial use. Therefore, for the practical application of genome-edited seed-fertile crops, varieties that do not contain foreign genes through genetic segregation are usually produced. Indeed, many countries have established guidelines dictating that such genome-edited crops are to be treated in the same manner as those produced by conventional breeding (i.e. not regulated as GMOs or on a case-by-case basis)^1^. In practice, commercial use of genome-edited soybeans and tomatoes has been implemented in the US and Japan, respectively^2^. In contrast, in the case of vegetatively propagated crops such as potato and banana, removal of foreign genes by crossing is difficult. Therefore, genome-edited lines of these crops are being produced via the transient introduction of site-specific nuclease enzymes/genes. In both seed-fertile and vegetatively propagated crops, it is extremely important to determine whether foreign nucleic acids remain in the final genome-edited varieties from the perspective of GMO regulation.

In a previous study, we disrupted the *SSR2* gene, a toxic steroid glycoalkaloid biosynthesis enzyme gene in tetraploid potatoes, by genome editing using TALEN^3^. In that study, we infected potato stem explants with *Agrobacterium* carrying the TALEN expression vector and regenerated shoots under non-selected conditions. Among the regenerated lines, we found several lines in which the *SSR2* sequence had been edited by genome editing, but the partial foreign sequences on the T-DNA were not detected by PCR analysis.

There are three main methods for experimentally detecting foreign nucleic acids in plants. The first is the PCR method, which is the most standardized and commonly adopted method. Indeed, target genes can be detected with high sensitivity using DNA polymerase and oligo DNA (primer) for the DNA sequence to be detected. The second approach is the conventional Southern blotting method. This method can detect sequences that are homologous to the probe, and can also detect shorter DNA strands (several tens of base pairs) compared to PCR; however, this technique requires a large amount of genomic DNA, and the results are highly variable depending on the skill of the operator. The third approach is the *k*-mer method, which is a relatively new technique that uses high-throughput sequencing (also known as next generation sequencing, NGS) data. In this method, large amounts of short-read NGS data are obtained from genomic DNA extracted from genome-edited plants, and the number of short DNA fragments (called *k*-mer) on the vector sequence used for genome editing is counted. This is then compared with the counts in the wild type and statistically analyzed to verify whether the foreign nucleotides are still present. The *k*-mer method can detect short foreign DNA fragments regardless of the skill level of the operator, assuming a sufficient amount of NGS data. However, this method has only been reported for a limited number of crops, such as rice , wheat, and soybean, and not for vegetatively propagated plants such as potatoes^4,5^.

In this study, to determine the applicability of the *k*-mer method, we detected foreign DNA on genome-edited potatoes produced in our previous study that presumably do not retain foreign DNA. Based on our results, we confirm the usefulness of *k*-mer analysis for the detection of foreign genes in genome-edited plants, and we consider this technique to be one of the most promising evaluation methods for foreign gene detection in the regulation of genome-edited crops.

## Results

### Computational simulation

To demonstrate the utility of *k*-mer analysis in potatoes, we performed computational simulation analyses. First, the detection of a 20-nt insert using 20-mer analysis was simulated by performing 5000 trials with different NGS data depths. As shown in Table 1, we successfully detected almost all 20-nt insertions at depths greater than 15, and 100% of insertions at depths greater than 30. Next, *k*-mer analysis was performed using wild-type (WT) NGS simulation data generated using different seed values to confirm the rate of false positives. The number of false positives decreased with increasing *k*-mer length, and at the commonly used *k* = 20, 0.20 ± 0.54 false positives were detected per trial at × 30 depths. No false positives were detected at *k* = 23 and above with this depth (Table 2).

**Table 1:** Number of successful detections of foreign DNA fragments over 5000 iterations in the computational simulation with different NGS data depths.

NGS depth (x)	10	15	20	30
Number of accurate detections out of 5000 attempts (%)	4289 (85.8)	4940 (98.8)	4980 (99.6)	5000 (100)

NGS data were generated at each depth from randomly inserted pseudo genomic sequences with 20-nt sequences randomly extracted from the pRI 201-AN-GUS vector, and *k*-mer analysis was performed at *k* = 20. The number of trials that successfully detected the sequence at the 1% level (*G*-test) was counted.

**Table 2:** Average number and standard deviation of false positive hits in the computational simulation with different NGS data depths.

*k*-mer	x10	x15	x20	x30
16	7.49 ± 6.75	6.97 ± 6.40	6.15 ± 5.73	7.35 ± 6.69
17	2.77 ± 3.07	3.04 ± 3.23	2.32 ± 2.57	2.91 ± 3.24
18	0.74 ± 1.29	0.93 ± 1.41	0.81 ± 1.36	0.86 ± 1.38
19	0.27 ± 0.69	0.34 ± 0.95	0.45 ± 1.39	0.33 ± 0.78
20	0.12 ± 0.37	0.24 ± 0.85	0.36 ± 1.33	0.20 ± 0.54
21	0.01 ± 0.13	0.15 ± 0.63	0.27 ± 1.07	0.12 ± 0.39
22	0.01 ± 0.09	0.08 ± 0.42	0.21 ± 0.79	0.03 ± 0.16
23	0	0.02 ± 0.19	0.11 ± 0.46	0
24	0	0.01 ± 0.08	0.05 ± 0.22	0
25	0	0	0	0

False positive hits were counted using the NGS simulation data of the wild-type potato genome and pRI-201-AN vector sequence.

### *k*-mer analysis with real NGS data obtained from genome-edited potatoes

**Figure 1:**
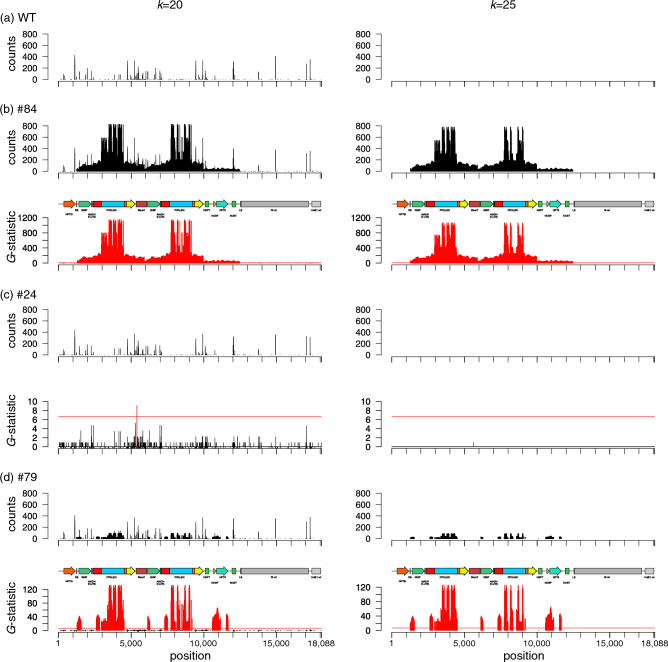
Detection of identical *k*-mers between the actual genome-edited potato genome and vector sequences. Data obtained from the wild-type (WT) (**a**) and selected genome-edited potato lines #84 (**b**), #24 (**c**) and #79 (**d**) are shown for *k* = 20 (left) and *k* = 25 (right). Line #84, in which transgenes were detected by PCR in a previous study, was used as a positive control. The results of the counts and *G*-statistic values (against the WT) at each position in the pYS_026-SSR2_C vector sequence used in genome-editing are shown in the vertical plots. The red horizontal line corresponds to the 1% significance level (*G*-values > 6.634) and the vertical plots exceeding this line are shown in red. Plasmid maps are also shown above the results. The results for other genome-edited lines are shown in Fig. S1. The *k*-mer analysis results obtained by repeated NGS experiments are shown in Fig. S2.

We obtained NGS data from genomic DNA extracted from the WT and genome-edited potatoes as shown in Table S2. The NGS data were obtained for all lines with a data volume of more than × 30 depth. In transgenic line #84, significant signals were observed in a wide region of T-DNA of binary plasmid pYS_026-SSR2_C at both *k* = 20 and *k* = 25 (Fig. 1). In the other genome-edited lines (#24, #26, #75, #117, #118, and #121), only a few or no significant signals were detected in the 20-mer analyses. We did not detect any significant peaks in the 25-mer analyses in these lines (Fig. 1, Fig. S1). Information on the detected peaks is summarized in Data S1 and S2. Surprisingly, in strain #79, fragmented peaks in the T-DNA region were detected in both the 20-mer and 25-mer analyses. NGS data acquisition and *k*-mer analysis were repeated to confirm whether the detected peaks were false positives or not. In transgenic lines #84 and #79, almost all peaks were commonly detected between the repeated experiments (Fig. S2 a,c). The detection of *k*-mer was confirmed in strain #117, specifically in the second NGS analysis (Fig. S2 b,d). Reads containing the detected *k*-mer were extracted from second NGS data for #117, and matched almost perfectly with other plasmid DNA used in our laboratory but showed low homology to the vector used for genome editing (Table S3). In the other genome-edited lines, no peaks were commonly detected between the repeated experiments (Fig. S2). The positively detected *k*-mer sequences are shown in Data S1 and S2, and the results of all the *k*-mer analyses are summarized in Data S3.

### Validation of *k*-mer analysis using genomic PCR

**Figure 2:**
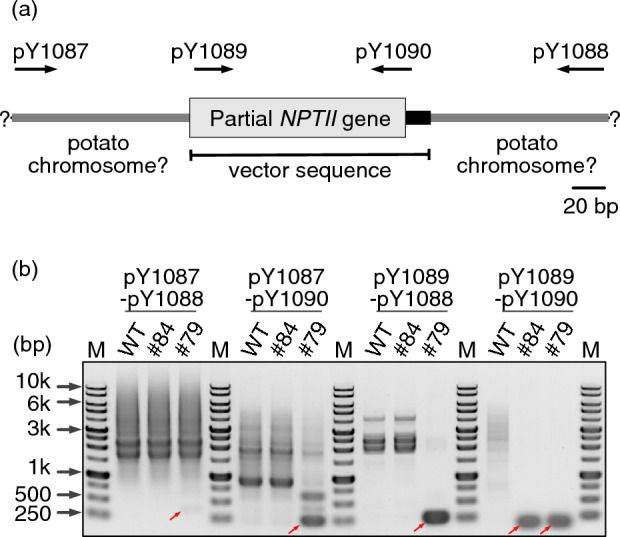
Validation of a T-DNA fragment within the potato genome by PCR. (**a**) Schematic illustration of a small partial T-DNA fragment insertion site in a genome-edited potato, #79, estimated from NGS reads. The nucleotide sequence information is shown in Fig. S3. Primers used in the PCR experiment are also shown. (**b**) Detection of a small partial T-DNA fragment insertion by PCR. Purified genomic DNA extracted from wild-type (WT) and genome-edited potato lines #84 (containing whole T-DNA sequence) and #79 were used as the PCR template. Amplification of DNA fragments of the expected size is indicated by the red arrows. M; ExcelBand 1 KB (0.25–10 kb) DNA Ladder (SMOBIO Technology, Inc.). The original gel image is presented in Supplementary Figure 4.

To confirm whether the peaks detected in the *k*-mer analysis were inserted into the potato genomic DNA, PCR experiments were performed. Among the peaks detected, a small DNA fragment detected in line #79 (approximately 150 bp, from vector position 11,534 to 11,667) was tested. Reads containing 20-mer sequences at both ends (11,534–11,553 and 11,667–11,686) were extracted from the NGS data and alignment was performed. An NCBI BLAST search of the adjacent sequences at both ends showed that the sequences corresponded to potato chromosomal sequences (Fig. 2a, Fig. S3). However, as homologous sequences were found in multiple chromosomes, the chromosome corresponding to these sequences could not be determined. We performed PCR using primers corresponding to the adjacent potato chromosome and vector fragment sequences, and amplification of a DNA fragment of the expected size was specifically confirmed in #79 (Fig. 2b).

### Evaluation of chromosome copy numbers in genome edited potatoes

**Figure 3:**
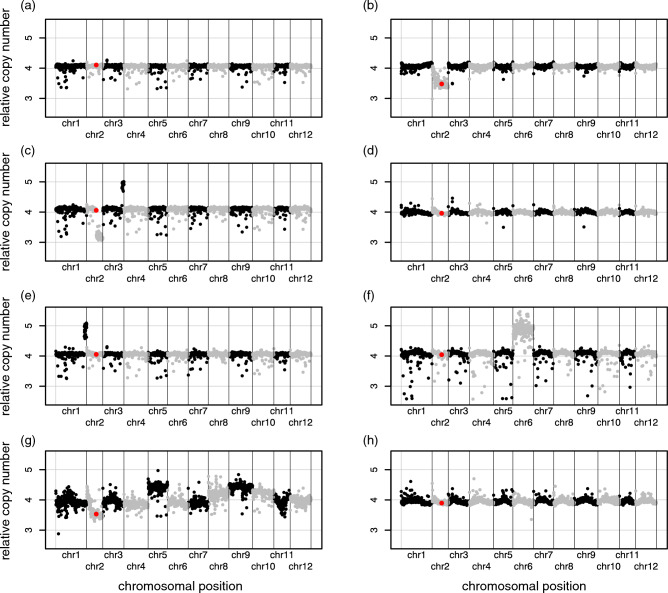
Chromosomal copy number validation of genome-edited potatoes generated by *Agrobacterium*-mediated TALEN transient expression. The relative copy number on the y-axis is plotted against 250-kbp chromosomal bins on the x-axis in genome-edited potato lines (**a**) #24, (**b**) #26, (**c**) #75, (**d**) #79, (**e**) #84, (**f**) #117, (**g**) #118 and (**h**) #121. A 250-kbp bin containing the *SSR2* gene (the target of the genome editing) on chromosome 2 is highlighted in red. As the potato cultivar used in this study is tetraploid, four genomic copies were expected.

Chromosome copy number analysis was performed using the NGS data used for the *k*-mer analysis. The variations in plots at each chromosome, which may be caused by variations in the NGS data, were confirmed in all genome-edited lines (Fig. 3). We detected obvious changes in the copy number of chromosome fragments in several genome-edited lines, e.g. in chromosome 2 of #26, chromosomes 2 and 3 of #75, chromosome 1 of #84, chromosome 6 of #117, and chromosome 2 of #118.

## Discussion

In addition to the *k*-mer method, several methods have been proposed to detect foreign genes in plants using NGS data. FED^6^ and CTREP-finder^7^ are mapping-based foreign DNA detection programs that require reference genome information. Recently, potato reference genomic sequences have been reported^8,9^; however, because of the variation in different cultivars, it is impossible to use these programs if the genome information of the cultivar used for genome editing is not publicly available. In addition, both programs are available online only with no open-source code, which does not ensure the reproducibility of the analysis. The *k*-mer analysis, for which the source code is available on GitHub, is advantageous because it does not require reference sequences but only NGS data of genome-edited and WT lines, and the nucleotide sequence of the plasmid DNA used for genome editing. INSIDER is a *k*-mer signature-based program that identifies DNA sequences potentially originating from foreign genes^10^. This program is available via GitHub, but the problem is that the shortest foreign gene length required for detection is 2 kb, much longer than that required for the *k*-mer method. Compared with these programs, the *k*-mer method is expected to be robust in detecting foreign genes, but, to date, its use has been reported only for seed-fertile crops such as rice, wheat, and soybean^4,5^. In the present study, the applicability of the* k*-mer method was evaluated in an important crop, potato, which is a vegetatively propagated crop with a complex heterogeneous tetraploid genome.

We first performed a computer simulation to confirm the feasibility of applying *k*-mer analysis for detection of exogenous nucleotides in the potato genome. The results showed that using NGS data at a depth of × 30 the genome size, it is possible to reliably detect foreign genes even with the insertion of extremely short DNA fragments, such as 20-nt in length (Table 1). The cost of acquiring NGS data has been decreasing annually. Although it depends on the NGS platform used, it is estimated that × 30 depth data acquisition (approximately 100 Gbp) for a tetraploid potato genome would cost approximately only 700 USD using Illumina HiSeqX^11^ and USD 1000 using Illumina NovaSeq 6000^12^. We also examined the false positive hits of *k*-mer analysis in potatoes by computational simulation. In the analysis for *k* = 23 and 24, false positives were confirmed for × 15 and × 20 depths, while none were confirmed for × 10 and × 30 (Table 2). This may be because the statistical power of the differential detection was too low for a × 10 depth. It is thought that setting a *k*-value of an appropriate length will reduce the detection of false positives to a certain level. In fact, no false positives were detected in the × 30 depth NGS data simulation with *k*-values greater than 23 when the pRI-201-AN-GUS sequence was used. Short *k*-mer fragments have a high probability of being present in both the vector used for genome editing and the host genome. Therefore, we found that setting a small value for *k*-mer can result in a coincidentally significant difference between the number of reads in the NGS of the sample and the WT, which could be detected as an exogenous sequence. Thus, the *k*-mer value should be set considering the possibility of detecting false positives and the probability of missing short DNA fragments. Previous research by Ito et al. showed that false positives in rice were detected at *k* = 25 or higher^4^. This may be due to an overestimation in the false positive rate as these authors performed computational simulation using rice genome sequences with inserted foreign nucleotides.

Next, we performed *k*-mer analysis using real NGS data obtained from genome-edited potato plants using 25-mer in addition to 20-mer as used in previous studies. The genome-edited potatoes were previously generated by *Agrobacterium*-mediated transient expression of TALEN^3^. In transgenic line #84, in which the amplification of the T-DNA region was previously confirmed by PCR tests, remaining foreign nucleic acids were clearly detected by *k*-mer analysis, indicating that foreign genes can be detected in the potato genome using *k*-mer analysis (Fig. 1). For several other lines (#24, #26, #117, and #118), only sporadic peaks were identified in the *k* = 20 analysis (Fig. S1). Careful observation of the analysis data for the detected peaks revealed that all of the sporadic peaks corresponded to the sequences detected in the WT genomes as well as the genome-edited lines (Data S1 and S2). This suggests that these peaks were false positives, possibly caused by the multiple comparison tests.

One genome-edited line, #79, showed fragments in the T-DNA region that were significantly detected. In general, *Agrobacterium* transports full T-DNA in binary plasmid vectors in plant cells; however, the rare introduction of sequences outside of T-DNA or partial T-DNA has been reported in some other plants such as *Arabidopsis thaliana*^13^, and the DNA fragment detected in the *k*-mer analysis of line #79 is also considered to consist of foreign nucleotides introduced by *Agrobacterium*. To confirm that the unique foreign DNA fragments detected in this line were stable insertions into the potato chromosome, we rechecked the NGS data. Several reads were identified in which the detected *k*-mer was bound to a sequence that appeared to be a potato chromosome sequence (Fig. 2a, Fig. S3).

To evaluate the results from the NGS data, we performed PCR using genomic DNA as the template with primers designed inside and outside the approximately 150-bp DNA fragment (part of coding sequence for *NPTII*) as detected by the *k*-mer analysis. No specific DNA amplification was observed in the negative control, i.e. a sample with the WT genome as the template. In contrast, in the PCR using the inner primers, specific bands were detected in the genomic DNA from lines #79 and #84 (i.e. transformants with full-length T-DNA). Reactions combining inner and outer primers, or outer primer pairs, showed specific amplification in line #79, indicating that a DNA fragment of approximately 150 bp was inserted into the chromosome (Fig. 2b). Schouten et al. found a tiny 50-bp fragment originating from a central part of the T-DNA in the genome of *Arabidopsis* transformants generated by *Agrobacterium*-mediated floral dip transformation by NGS^13^. As these authors analyzed *Arabidopsis*, for which highly accurate genomic information is available, they could detect this small DNA fragment by mapping NGS reads to the genomic sequence. In contrast, for potatoes, which retain a complex tetraploid genome, a similar approach is difficult, and *k*-mer analysis is more suitable for the detection of foreign nucleotides.

We also analyzed the NGS data for the transgenic potato on the flanking sequences of other DNA fragments containing part of right border and 35S promoter sequence. We confirmed NGS reads containing the vector sequence (1268–1288), and found the sequences homologous to the *Agrobacterium* genome as adjacent sequences. Similarly, by extracting the adjacent sequences from the NGS data, a portion of the DNA fragment was suggested to have been inserted into the potato chromosomal genome via* Agrobacterium* genomic DNA integration, as shown in Fig. S3. The co-incorporation of T-DNA and *Agrobacterium* chromosomal DNA into plant genomes has been previously reported in the model plant *A. thaliana*^14^; however, as far as we know, such a phenomenon has never been reported in potatoes. This finding strongly suggests that the possibility of such non-canonical gene transfer needs to be considered when producing genome-edited crops through transient or stable transformation using *Agrobacterium*.

In line #117, specifically in the second NGS analysis, the positive *k*-mer were detected in the narrow position shown in Fig. S2 with slightly lower *G*-values compared to transgenic lines #79 and #84. Detailed evaluation of the NGS data revealed that the reads containing the *k*-mer in line #117 almost completely matched a binary plasmid, pBYR2HS^15^ or its derivatives, which was not used for the generation of genome-edited potato line #117. If the *k*-mer sequence is indeed derived from the genome-editing vector, the sequence adjacent to the *k*-mer should be a sequence from the plant genome, yet a BLAST search did not match the adjacent sequence of the detected *k*-mer to a plant-derived sequence (data not shown). In our laboratory, pBYR2HS and its derivative plasmids were used for producing useful triterpenoid compounds in *Nicotiana benthamiana*^16^. Therefore, the peaks detected in the *k*-mer analysis in the second NGS analysis of #117 were probably caused by contamination of plasmid DNA during the extraction and purification of genomic DNA from genome-edited potato plant. This is supported by the fact that a similar *k*-mer peak was not detected in the first NGS dataset. Thus, while analysis using NGS data can detect foreign genes with high detection sensitivity, it is susceptible to environmental contamination, and, therefore, samples must be handled carefully during the extraction and purification of genomic DNA and during the NGS analysis. However, the number and position of the detected false positive peaks varied somewhat among the non-preprocessed and preprocessed data, yet there was no effect on the detection of foreign genes, such as those detected in lines #79 and #84 (Data S1 and S2).

We also preliminarily tested for chromosome copy number variation in the genome-edited potatoes using the NGS data used for the *k*-mer analysis, although a greater NGS data volume would be required for more accurate testing. No clear chromosomal changes were observed in several genome-edited lines, but the results of lines #26, #75, #84, #117, and #118 suggest an increase or decrease in the chromosome number (Fig. 3). Previous studies have reported that simple regeneration can change the number of chromosomes in the potato genome^17^. Owing to the small sample size, it is difficult to determine whether this change in chromosome number was because of genome editing or the regeneration process. However, some lines (#26, #75, and #118) showed a decrease in the number of chromosome 2 on which the *SSR2* gene—the target of genome editing in this study—is located, thereby strongly suggesting that genome editing caused the observed chromosome-level changes. We were unable to detect phenotypic changes under aseptic culture conditions even in the lines in which chromosomal changes were observed (data not shown); it is possible that some phenotypic changes could be observed under actual field conditions, but if the changes were associated with cultivation problems, such defective lines would be omitted from selection during the breeding process.

In summary, we investigated the *k*-mer method as a new technique for detecting foreign genes in the potato genome. Our results show that this method can accurately detect foreign genes in genome-edited potatoes when using a sufficient amount of NGS data and selecting appropriate *k*-mer values. Although this study analyzed genome-edited potatoes developed using TALEN, the *k*-mer method can theoretically be applied to plants developed using other genome-editing tools such as CRISPR-Cas9. While the PCR-based method, which is most commonly used, can detect foreign DNA with high sensitivity, it is more difficult to comprehensively detect shorter DNA fragments in the order of 100 s of bp as were successfully detected here. Thus, we propose that the PCR and NGS (*k*-mer) methods can be used in combination to more reliably detect foreign nucleic acids in genome-edited crops.

## Methods

### Computational simulations of *k*-mer analysis in the potato genome

The double monoploid potato genome sequence (DM_1-3_516_R44_potato_genome_assembly.v6.1.fa.gz) was obtained from the Spud DB Potato Genomics Resource (http://spuddb.uga.edu/dm_v6_1_download.shtml). This sequence information was quadrupled to generate pseudo-tetraploid potato genome information. A mimicked transgenic genome sequence was generated by inserting a 20-nt sequence randomly extracted from sequence information of the binary vector pRI 201-AN-GUS (TaKaRa Bio, Japan) as exogenous nucleotides into a random position in the potato genome. To estimate the depths of NGS data required for the *k*-mer analysis of potatoes, NGS data with different depths were generated by simulation, as follows. We generated NGS simulation data at depths of × 10, × 15, × 20, and × 30 from the respective sequence information using the “Sandy” tool (https://github.com/galantelab/sandy). The *k*-mer analyses^4^ (https://github.com/taitoh1970/kmer) were performed using 10 WT simulation datasets generated from the pseudo-tetraploid potato genome with different seed values and 500 mimicked transgenic genome simulation datasets. For each of the 5000 analyses (10 WT × 500 transformants), the number of successfully detected inserted foreign DNA sequences was counted and used as the detection accuracy for the *k*-mer analysis. Next, to confirm the false positive rate, 9900 k-mer analysis (100 WT × 99 WT) were performed on WT genomes generated at different seed values to determine the number of *k*-mer detections when the pRI-201-AN vector sequences were used.

### Plant material

Genome-edited and WT potatoes (cultivar Sassy) were subcultured on Murashige and Skoog (MS) medium containing sucrose solidified with agar as previously described^3^. Genome-edited lines #24, #26, #75, #79, #117, #118, and #121, in which PCR amplification of partial T-DNA region was not confirmed in the previous study^3^, were used as the samples. Transgenic genome-edited line #84 was used as the positive control.

### Analysis of the potato genome using the *k*-mer method on biological samples

The genomic DNA from nine samples (WT and eight genome-edited lines) were extracted and purified using the NucleoBond HMW DNA kit (TaKaRa Bio, Japan) and sequenced via the Illumina HiSeq X (for the first NGS data) and NovaSeq6000 (for the second and third NGS data) platform. The FASTQ sequence data are available from the DDBJ Sequence Read Archive under the following accession number: DRA015756. Raw Illumina reads (non-preprocessed data) and trimmed reads using “fastp” (preprocessed data)^18^ with the parameters –detect_adapter_for_pe -q 30 -n 10 -l 20 were used for the *k*-mer analysis^4^. The analysis was performed at *k* = 20 or 25. In some NGS data that were confirmed positive in the *k*-mer analysis, NGS reads containing boundary sequences of foreign nucleotides were extracted using “seqkit”^19^. NCBI-BLAST or alignment analysis was also performed on the extracted sequences to confirm that the detected *k*-mer sequences were integrated in the plant genome. For repeat experiments, genomic DNA was extracted from the same potato plant lines as the first NGS analysis and provided for the second and third NGS analyses.

### Validation of foreign nucleotide insertion in the potato genome using PCR

Genomic PCR reactions were performed as follows. Each 50 μL PCR mixture contained 25 μL of 2 × PCR Buffer for KOD FX Neo (TOYOBO, Japan), 1 μL of 10 μM forward and reverse primers, and approximately 100 ng of purified genomic DNA. The PCR mixture was incubated at 94 °C for 2 min, followed by 35 cycles of denaturing at 98 °C for 10 s, annealing at 60 °C for 30 s, and extension at 68 °C for 30 s. The PCR solutions were subsequently analyzed by agarose gel electrophoresis. Table S1 lists the primer sequences used in this study.

### Evaluation of chromosome copy numbers

Chromosome copy number analysis was performed according to previous studies^17^ using NGS data obtained during the *k*-mer analysis with minor modification. Potato chromosome sequence information was clipped to 250 kbp each, and reads from the NGS data were mapped to each sequence using “bwa-mem2” (https://github.com/bwa-mem2/bwa-mem2). The number of reads binned to each sequence was then counted using “SAMtools” software^20^ and standardized for chromosome copy dosage using the counts from the WT sample.

## Supplementary Information

Supplementary Information.

Supplementary Figure S4.

Dataset S1.

Dataset S2.

Dataset S3.

## Data Availability

The datasets generated during the current study are available in the DDBJ Sequence Read Archive repository, DRA015756.
