# Supplementary material for: Foreign DNA detection in genome-edited potatoes by high-throughput sequencing
Source: Sci Rep. 2023 Aug 9;13:12246. doi: 10.1038/s41598-023-38897-x (PMC10412624; doi:10.1038/s41598-023-38897-x)
Supplement: Supplementary file 1 — Supplementary Information. [file 41598_2023_38897_MOESM1_ESM.pdf]

Supplementary information

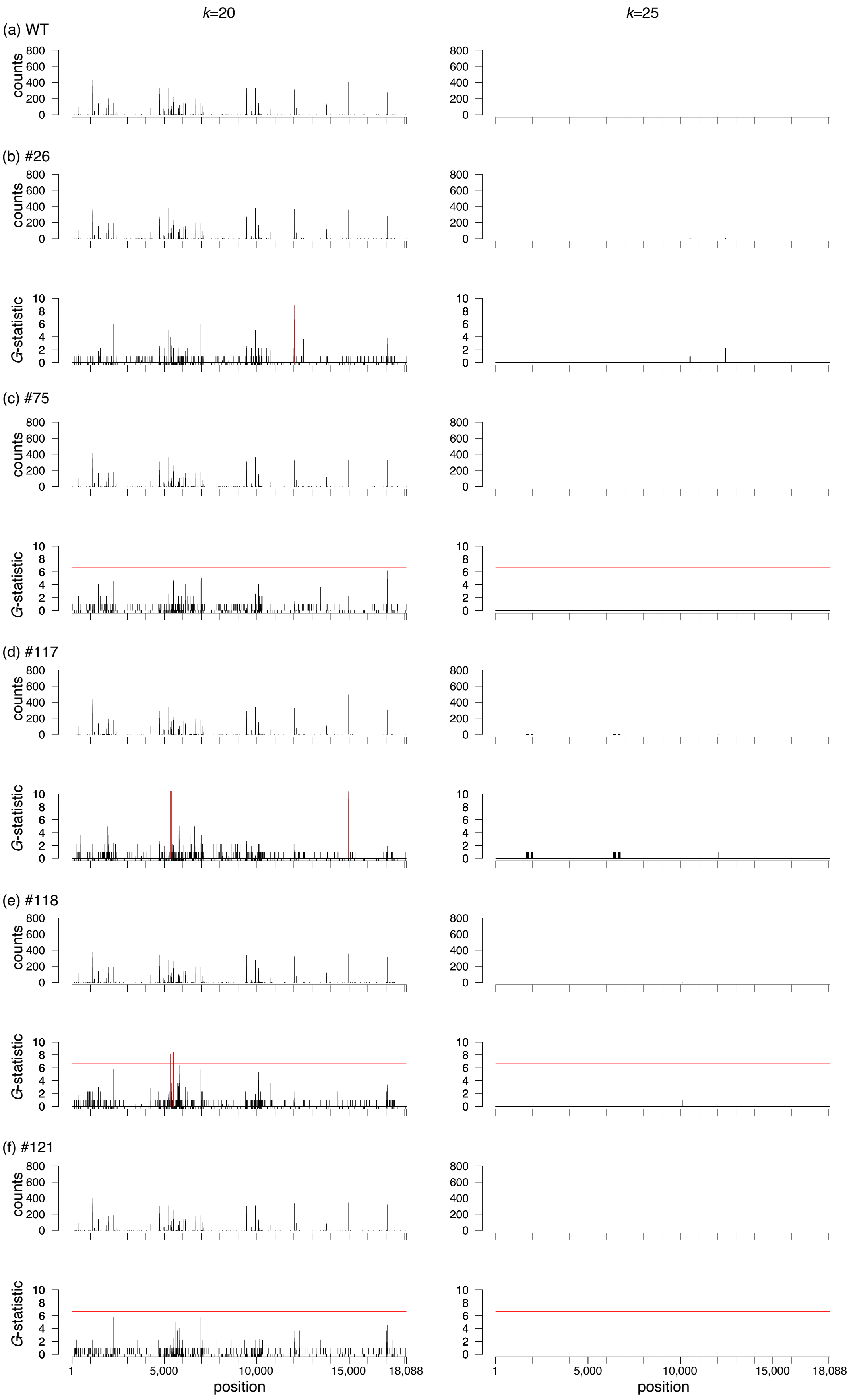

Figure S1. Detection of identical *k*-mers between the actual genome-edited potato genome and vector sequences.

Data obtained from the wild-type (WT) (a) and genome-edited potato lines not shown in Figure 1, #26 (b), #75 (c), #117 (d), #118(e), and #121 (f) are shown for $k = 20$ (left) and $k = 25$ (right). The results of the counts and *G*-statistic values (against the WT) at each position in the pYS_026-SSR2_C vector sequence used in genome-editing are shown in the vertical plots. The red horizontal line corresponds to the 1% significance level (*G*-values > 6.634) and the vertical plots exceeding this line are shown in red. The results obtained by repeated NGS experiments are shown in Figure S2.

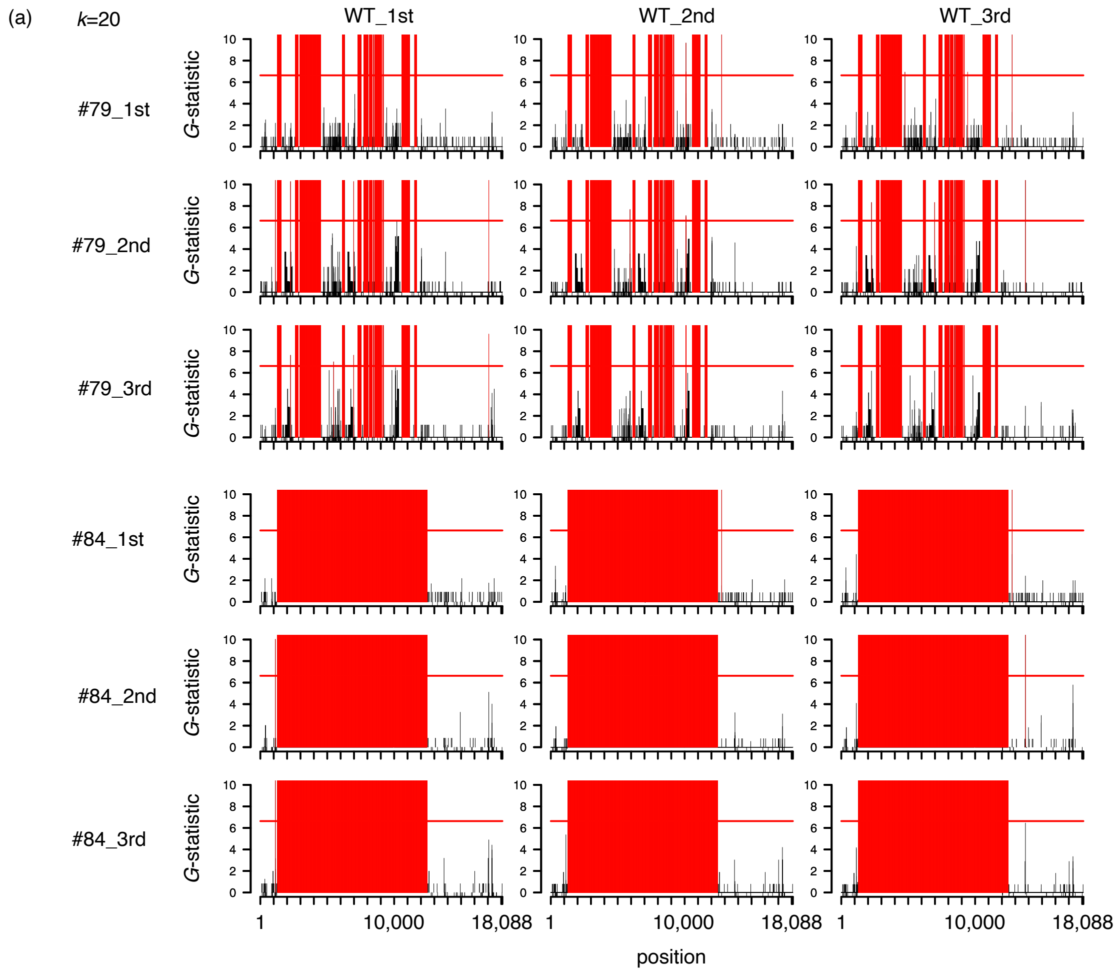
(a)
k=20
WT_1st
WT_2nd
WT_3rd
#79_1st
#79_2nd
#79_3rd
#84_1st
#84_2nd
#84_3rd
G-statistic
1
10,000
18,088
position

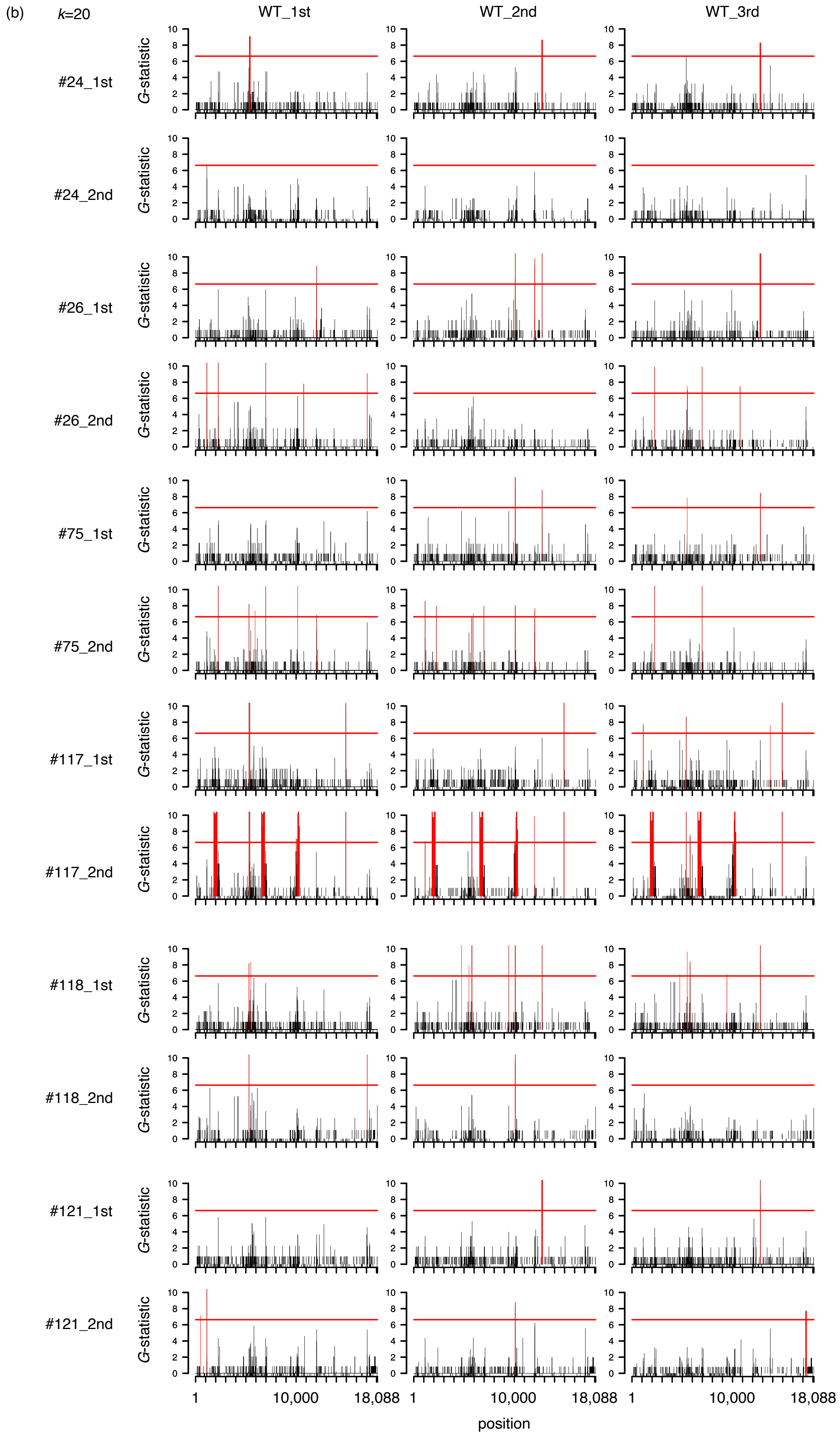
(b)
k=20
WT_1st
WT_2nd
WT_3rd
#24_1st
#24_2nd
#26_1st
#26_2nd
#75_1st
#75_2nd
#117_1st
#117_2nd
#118_1st
#118_2nd
#121_1st
#121_2nd
G-statistic
1
10,000
18,088
position

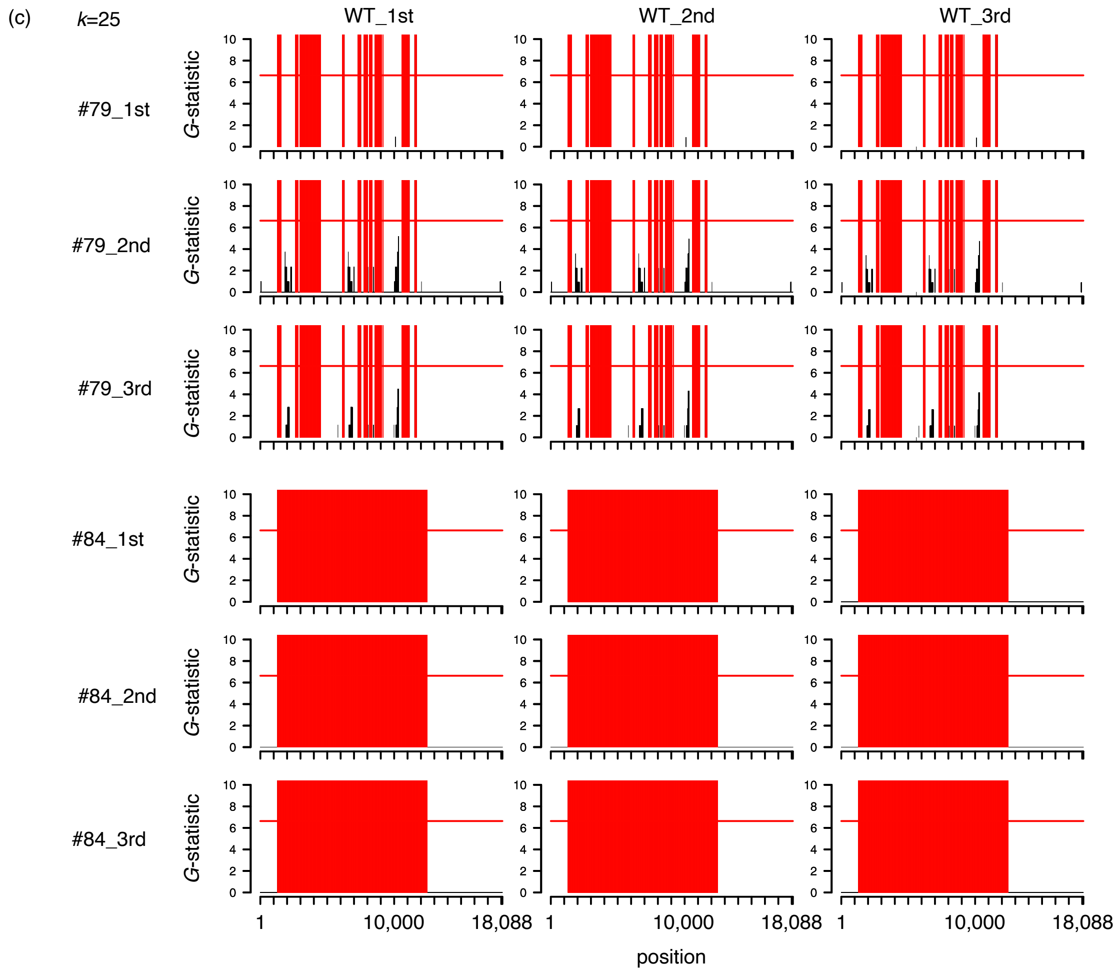

(c)
k=25
WT_1st
WT_2nd
WT_3rd
#79_1st
#79_2nd
#79_3rd
#84_1st
#84_2nd
#84_3rd
G-statistic
1
10,000
18,088
position

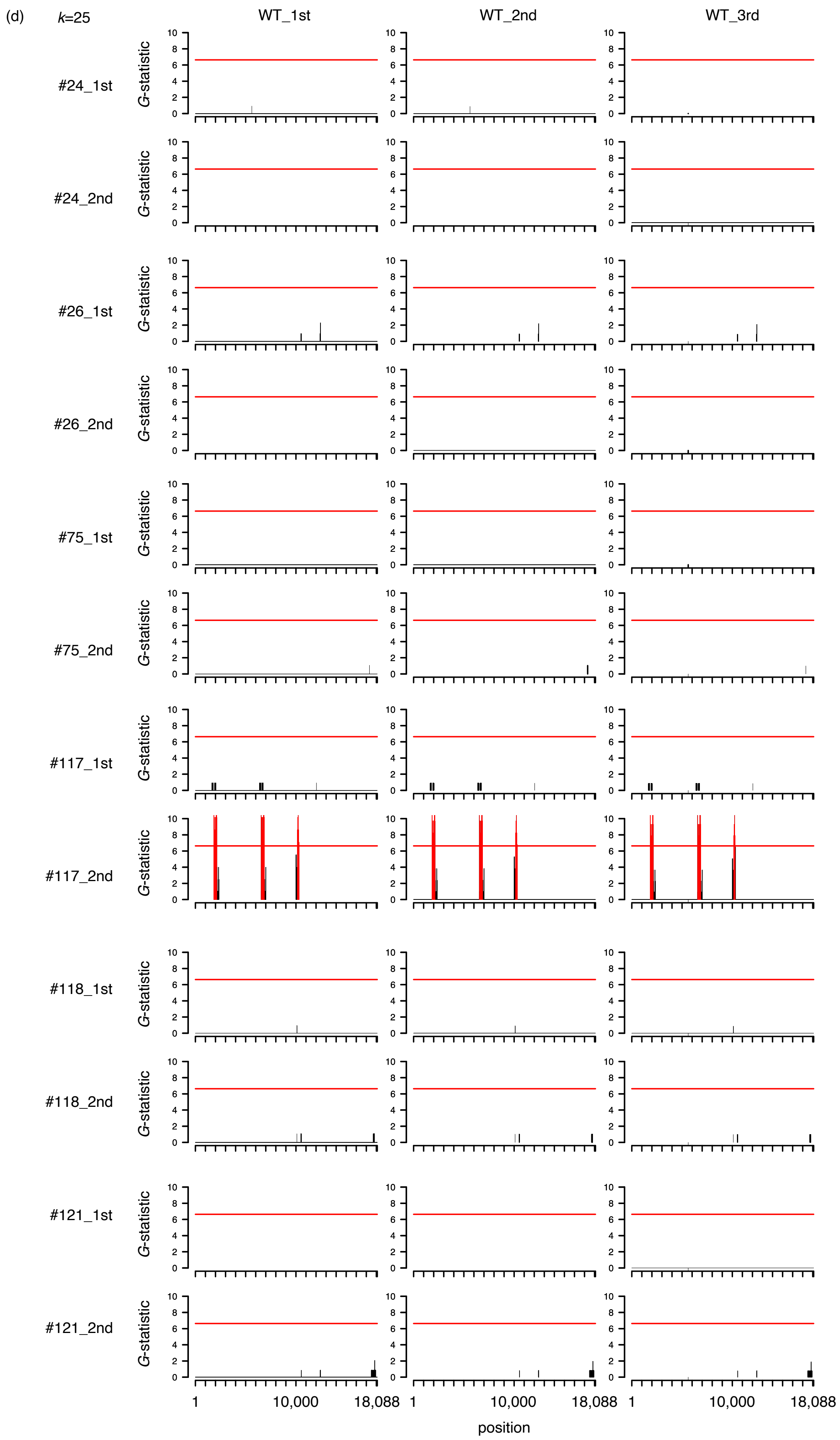
(d)
k=25
WT_1st
WT_2nd
WT_3rd
#24_1st
#24_2nd
#26_1st
#26_2nd
#75_1st
#75_2nd
#117_1st
#117_2nd
#118_1st
#118_2nd
#121_1st
#121_2nd
G-statistic
1
10,000
18,088
position

Figure S2. *k*-mer analysis using repeated real NGS data.

The result of *k*-mer analysis using repeated NGS data obtained from the wild-type (WT) and genome-edited potato lines are shown for k = 20 (a, b) and k = 25 (c, d). The result of *k*-mer analysis for genome-edited lines assessed as transgenic lines (#79 and #84) by *k*-mer analysis first using NGS data are shown in (a) and (c). The results of *k*-mer analysis for genome-edited lines assessed as having no transgenes (#24, #26, #75, #117, #118, and #121) first using NGS data are shown in (b) and (d). *G*-statistic values over 10 are omitted, and the raw data are shown in Data S1.

(a)

5′cgtgacaaagttggtatcatagccctaggttcattaatctcattgtatcgaaacgaggct
agtagagtcttgcggaacggtacgaagacgtgactatgactgacttgatatatACATAGCGT
TGGCTACCCGTGATATTGCTGAAGAGCTTGGCGGCGAATGGGCTGACCGCTTCCTCGTGCTT
TACGGTATCGCCGCTCCCGATTCGCAGCGCATCGCCTTCTATCGCCTTCTTGACGAGTTCTT
CTGAGCGGGACTCTGGGGTTgatttggttatgctgaatatgactgacttgatataatgttag
gcatgacttggttgtcccccctattatgttgtgcttaatggtaatgctaagtcggtaactcta
gagatcccg3′

(b)

5’gggtggtggtgatttgtaaactggtgcgggatgataaggtgttggtgaagggtggtatggc
ttctttggtggtgggggagatgagtattgataattagctgagctttcggaagctaagctaag
tgacaagaaagacgacctgcggaccaaacaaggcaaacatcttccgcaatgagtgtagatgc
gtccggctcggccgcagctcattgattccgaggccagctctatcaggagctcttgattttg
gacccgagcatcatcgtgcccttggtcttgagctggtgatggtttcgaaggcgaattcgcag
ttcgaggctgtcaaacaaaccctgcaacatttcgatgaacttgggatgtgtcgtccgagtgc
tgagactaccgcgccgcaacgtcgtcaagggaatattgagactgataaagccgttctctggc
gtatgcaggtcgaccacttcgccatcattgtagcggtccagtagtgtgacggccaaagcagc
gaacgctatgaagacaatggagcgcggtcgctgagaaatttcagctgtcgcccctgaggttc
tcgggtcagcgttcagagagagcaatctgtttccagcgcctagcctgctcgcgaatttttcc
tgcttagcaacatcacctctgacgcggttcgacacgtatacgggtttctccaggccgtctgc
ctccatgtcgatcgccccgatcagactgtcgagaccgccggacaacagtgcgacgcaggttt
cgggacgcgatccgataacctgtttcggcgggcgtgggtggtatccgccgcatttccagaa
gccatcacgagttccctcgcaccgggtgatgaatatcgtcacttctccagatagcggtcaag
ctcgcgatatcttgtcaaagaacttccagtatgccgcgaatgcaccggcggcaatcagatca
agaatgaaccacgacgaccggctcaaatgcaccgggataagcgggttaaacagcaacgccac
gcaaatgaagccgaacacccaacccgtaaacgccttgtgctgtccgtactcgatgtaagcga
agaacgctgcgaatgcagtgatgacgaggcgcaacagcgtgtagtagccatagggcaacgga
aatatagcaatcactaatgctactgctggaaggaggatgatgtggcgtggaaatatggtcac
gtaagcccccaaacatgtctatgaaaatcatctaattcacaaacataagtttggaaagcag
gcagatacaatctcttgtggagtacgctgcgctacaagtcagcagtgcggagcgggatgact
aagccttattgtcaaggatatacgctgtatatgccgcttcaactcgatgaacgacggctttc
attaggtctttaaggatcacgatatgttcgtcgtcaatcacgacatcttttccatcattgtc
gcgcccgctgcgatgcacgaggtcatgccgcatcgtgataatgtctagcatttctttctcgt

gattttcgctctcaaacaagttcattttcagtacggctttgtaaaagacggccacgctggct
aagtcgtgaaacgagaaacgctgcaaatagttcttgcttgcagtcgccacatagttggggtc
cttggcgatgtcgataagtttaggttgattgttccgaagggcggggttgcaccgacgagcg
caagcagtttgtcgctatcgttggtgacgatcttgatgagccggtcgctcaagtacgcctca
agtgaaataacatgctggagcgagatcattttgtggcacgctcggccaggcactcgctgcac
ctgtcaagctggcggacggcagtaccgacctttccatcgatcaggagaagttccacgaccag
tttgctcacgatgtgggcgtagacaacgctgcgctgatggcggccgggcagcggcccatcac
tgaggcggcactgacggaaaagtcaggcaagccggcctggaaggcgctgccgtcctacttcg
tctacggtgatggcgacaagaacataccggccgaggcactcgccttcatggctgaacgggca
ggctcaaagcacacagtcgtcgcaaaaggtgcctctcacgtggtgatggtctcgcagccaga
ggtggtcgcagcccttattgaagaagcggcgaactaggctcgttcccgcgcagaagaccggc
tcctgcggtcggtcttctgcggaacgagacgggatcactcgttcggatcgcttcaaggcgtt
ggaagcgtagacttccattactttcatcaacgaaaatccgatcaaaaaataatttgttcagt
gtagtgaaagacgtccaagcctagactggatttgcgccataaggcgtctaataattcggcta
gtggagaaggaaaatcgcaagctcccgtgcgattgctcctcgatcctcgcaaccgaccgttt
atcaaccagacagtgtcaggagttgtcacgcctttgttactagaaggccaTCAAACACTGAT
AGTTTAAACTGAAGGCGGGAAACGACAATCTGATCCTGGCGAAAGGGGGATGTGCTGCAAGG
CGATTAAgttgggtaaCGCCAGGGTTTTCCCAGTCACGACGTTGTAAAACGACGGCCAGTGC
CAAGCTTGCATGCCTGCAGGTCCCCAGATTAGCCTTTTCAATTTCAGAAAGAATGCTAACCC
ACAGATGGTTAGAGAGGCTTACGCAGCAGGTCTCATCAAGACGATCTACCCGAGCAATAATC
TCCAggaaatcaaataccttcccaagaaggttaaagatgcagtcaaaagattcaggactaac
tagttgatggtctcgtcatcgttaaagcaggtcacgatcgcgacgaaggctttcttggtcat
catctatactccggtcattcaatcaggaaatgcttgcgaggcgagcattccggttggccgcc
tggacgtccgggtcagggtcgagggtggcggaaatcaaggcttgtgtgtagcgctgttgcgg
attatcgaagacttgcgcaactgtaccgaattcgaccacctcacctttctgcatgaccatga
cgtagtctgcaaaatcgcgcacgaggggcaggtcatgggcgatgaagatgaaggcaattccc
atctctcggcgcagcttgtcgagcagcctcaccacctgcgcctgcaccgatacatcaagtgc
cgaaacggcctcgtcgcagacgatcagttgcggctcaagcgcaagtgcgcgagcaatggcga
tacgctgacgctggccaccggagaactgatgcgggtagcgacccatatgttcggcgacagc
ccgacccgttccagcaattctgcggcgcgtgcacgccacttcgccttgggcagaatctccgg
gtggatgacccaggcct3′

Figure S3. Predicted neighbor sequence of foreign nucleotides (partial *NPTII*) in genome-editing line #79 based on NGS data.

(a) Predicted neighbor sequence of a small DNA fragment (approximately 150 bp) from the vector position 11,534–11,667 in the potato genome. Underlined capital letters indicate the homologous sequence to pYS_026-SSR2_C, the TALEN expression vector used in the genome editing. The partial *NPTII* sequence in pYS_026SSR2-C detected in the *k*-mer analysis is highlighted in green. Sequences homologous to potato chromosomal sequences (exact loci are unknown) are shown by red letters.
(b) Predicted neighbor sequence of a DNA fragment from the vector position 1,268–1,581 in the potato genome. Underlined capital letters indicate the homologous sequence to pYS_026-SSR2_C, the TALEN expression vector used in the genome editing. The partial RB sequence is highlighted in light blue; the partial 35S promoter sequence is highlighted by dark blue; sequences homologous to potato chromosomal sequences (possibly located on chromosome 4) are shown by red letters; and sequences homologous to the *Agrobacterium* chromosomal plasmid are highlighted in yellow.

Table S1. Primers used in this study.

Name	Sequence(5' to 3')	Note
pY1087/11534N-For	TGACAAAGTTGGTATCATAGCCCTAGG	target: potato genome
pY1088/11667N-Rev	GATCTCTAGAGTTACCGACTTAGCATTACC	target: potato genome
pY1089/11534-For	TAGCGTTGGCTACCCGTGATATTGC	target: vector sequence
pY1090/11667-Rev	CCGCTCAGAAGAACTCGTCAAGAAGG	target: vector sequence

Table S2. Summary of NGS data acquisition.

Sample	Sample ID	Total read bases (Gb)	Depth (x)	Preprocessed Total read bases (Gb)	Preprocessed Depth (x)	Note
Wild-Type Sassy	Sassy_NT	147.03	43.6	130.44	38.6	1st NGS data for WT
	Sassy_NT2	137.62	40.8	132.12	39.1	2nd NGS data for WT
	Sassy_NT3	129.60	38.4	123.73	36.7	3rd NGS data for WT
#24	Sassy_NS_24	147.62	43.7	131.96	39.1	1st NGS data for #24
	Sassy_NS_24_2	113.51	33.6	108.51	32.1	2nd NGS data for #24
#26	Sassy_NS_26	142.26	42.1	129.05	38.2	1st NGS data for #26
	Sassy_NS_26_2	141.16	41.8	135.39	40.1	2nd NGS data for #26
#75	Sassy_NS_75	143.08	42.4	129.25	38.3	1st NGS data for #75
	Sassy_NS_75_2	117.56	34.8	112.78	33.4	2nd NGS data for #75
#79	Sassy_NS_79	148.70	44.0	133.49	39.5	1st NGS data for #79
	Sassy_NS_79_2	137.67	40.8	131.63	39.0	2nd NGS data for #79
	Sassy_NS_79_3	104.04	30.8	99.65	29.5	3rd NGS data for #79
#117	Sassy_NS_117	145.69	43.2	131.90	39.1	1st NGS data for #117
	Sassy_NS_117_2	124.78	37.0	120.42	35.7	2nd NGS data for #117
#118	Sassy_NS_118	142.89	42.3	123.61	36.6	1st NGS data for #118
	Sassy_NS_118_2	119.40	35.4	114.94	34.0	2nd NGS data for #118
#121	Sassy_NS_121	142.70	42.3	118.80	35.2	1st NGS data for #121
	Sassy_NS_121_2	162.23	48.1	155.99	46.2	2nd NGS data for #121
#84	Sassy_NS_84	150.45	44.6	133.64	39.6	1st NGS data for #84 (positive control)
	Sassy_NS_84_2	166.23	49.2	159.34	47.2	2nd NGS data for #84 (positive control)
	Sassy_NS_84_3	170.61	50.5	163.18	48.3	3rd NGS data for #84 (positive control)

Sample : plant sample
Sample ID : NGS_data
Total read bases : Total number of bases sequenced (non-preprocessed raw data)
Depth: coverage of non-preprocessed raw data to tetraploid potato genome (844 Mbp)
Preprocessed Total read bases (bp): Total number of bases preprocessed by fastp (preprocessed data)
Preprocessed Depth: coverage of preprocessed data to tetraploid potato genome (844 Mbp)

Table S3. Detected plasmid sequence in #117 (second NGS dataset).

Vector position in pYS_026-SSR2_C	*k*-mer sequence	Read containing *k*-mer from #117 2nd NGS	Identity to pYS_026-SSR2_C (used for genome editing)	Identity to pBYR2HS-eGFP (possible contaminant)
1188	AACATGGTGGAGCACGACACACTTG	1	142/166 (85.5%)	151/151 (100.0%)
		2	138/152 (90.8%)	151/151 (100.0%)
		3	142/166 (85.5%)	151/151 (100.0%)
		4	135/180 (75.0%)	151/151 (100.0%)
		5	140/287 (48.8%)	150/151 (99.3%)
		6	149/149 (100.0%)	151/151 (100.0%)
		7	140/176 (79.5%)	151/151 (100.0%)
		8	148/149 (99.3%)	150/151 (99.3%)
2136	TCAAAGCAAGTGGATTGATGTGATA	1	142/166 (85.5%)	151/151 (100.0%)
		2	142/166 (85.5%)	151/151 (100.0%)
		3	148/151 (98.0%)	148/151 (98.0%)
		4	148/151 (98.0%)	148/151 (98.0%)
		5	140/287 (48.8%)	150/151 (99.3%)
		6	140/176 (79.5%)	151/151 (100.0%)
		7	151/151 (100.0%)	151/151 (100.0%)
10138	GCTAGCTTGTGTGCTTAAGTTTGTG	1	151/151 (100.0%)	151/151 (100.0%)
		2	151/151 (100.0%)	151/151 (100.0%)
		3	151/151 (100.0%)	151/151 (100.0%)
		4	151/151 (100.0%)	151/151 (100.0%)
		5	151/151 (100.0%)	151/151 (100.0%)
10326	GGAATATGATTAAAGATAAGGAATT	1	129/225 (57.3%)	151/151 (100.0%)
		2	140/179 (78.2%)	151/151 (100.0%)
		3	108/229 (47.2%)	151/151 (100.0%)
		4	137/176 (77.8%)	151/151 (100.0%)
		5	124/225 (55.1%)	151/151 (100.0%)

Identities were caluculated by local alignment program (water) in EMBOSS explorer (https://www.bioinformatics.nl/emboss-explorer/)

Data S1. Detected *k*-mer sequence for 20-mer analysis.

Data S2. Detected *k*-mer sequence for 25-mer analysis.

Data S3. *k*-mer analysis result.
