# Supplementary figures and images for: Foreign DNA detection in genome-edited potatoes by high-throughput sequencing
Source: Sci Rep. 2023 Aug 9;13:12246. doi: 10.1038/s41598-023-38897-x (PMC10412624; doi:10.1038/s41598-023-38897-x)

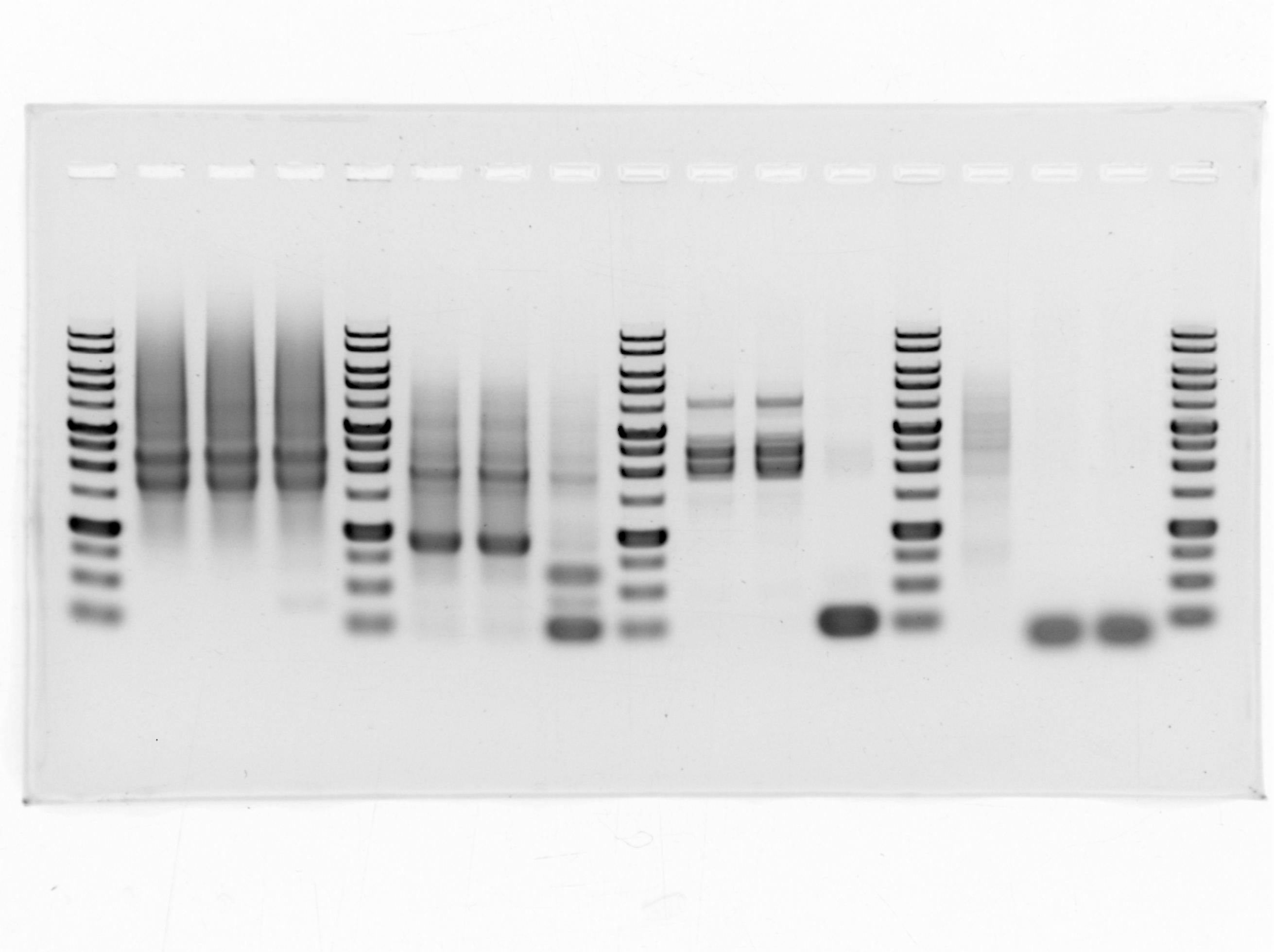

Supplement: Supplementary file 2 — Supplementary Figure S4. [file 41598_2023_38897_MOESM2_ESM.tif]
